# Living longer, working longer: the need for a comprehensive approach to labour market reform in response to demographic changes

**DOI:** 10.1007/s10433-014-0332-x

**Published:** 2015-01-17

**Authors:** Michal Myck

**Affiliations:** Centre for Economic Analysis, Szczecin, Poland

Increases in life expectancy over the last decades have been among the most salient reflections of changes in the quality of life in the developed world. In Germany, in 1990, the average life expectancy for men and women was, respectively, 72 and 79, while in Italy men could expect to live 74 years and women 80. By 2012, these numbers changed by about 6 years for men and 4 years for women in Germany and by 6 and 5 years in Italy. Substantial increases in life expectancy have been recorded in all EU countries, from Portugal to Estonia and from Cyprus to Norway. This growth reflects unequivocal success of medicine and health care provision and fundamental changes in the quality of life and lifestyle, and offers new opportunities for the growing groups of older populations.

However, when set against very low birth rates and low levels of economic growth, the increases have also called into question the financial sustainability of social security and public health care systems, and the potential generosity of welfare support for large groups of the population. It has long been recognised that increases in life expectancy, and the slower but equally important growth of healthy life expectancy, must be matched by adaptations of eligibility for public retirement pensions and by extended labour market activity. Continued economic development requires that increased proportions of the 50, 60 and 70 year olds be involved in the labour market, and that living longer be accompanied by working longer. There seems to be broad agreement that the process of extending working lives needs to involve all of the key stakeholders, i.e. both workers and their employers with an important supportive role of the government in areas of potential market failures. It also requires a comprehensive approach that combines labour market and retirement policies with tax policy and public health and welfare reforms.

The demographic changes that are occurring before our very eyes offer new opportunities for older individuals, insofar as many of them continue to enjoy healthy and active lives. At the same time these changes also present substantial challenges for many individuals who find themselves faced with new requirements at the workplace and difficult choices concerning care duties for older family members or support expectations among younger generations. It seems clear that as the proportion of the 50+ in the population grows, it becomes ever more important to understand the mechanisms which affect the quality of life of these groups of the population and which drive individual decisions in different areas of life, including activity in the labour market. Fortunately, as new survey and administrative data on older populations become available at national and international levels, researchers can examine the causes behind observed variation in different outcomes and they can address increasing number of key questions of academic and policy relevance.

The labour market activity of older people is the focus of the special section in this issue of the European Journal of Ageing, with each of the four articles in the section making its contribution to the current debate on increasing working lives. As argued by Sonnet et al. (2014), for example, this debate, apart from reforming retirement systems, has to address such areas as rewarding work, changing employer practises and improving employability of workers. The four papers in the special section fit well within this framework. Dal Bianco et al. (2014) and Achdut et al. (2014) examine different factors behind retirement decisions and retirement expectations. Belloni and Villosio (2014) analyse the role of training among workers aged 50+ for higher productivity, and thus, on the one hand, for attractiveness of older workers for employers, and on the other, financial reward of late career employment for the individuals. Finally, employer’s characteristics from the point of view of employability of older individuals are examined in the fourth paper (Tisch 2014). Two of the four papers (Dal Bianco et al. 2014; Belloni and Villosio 2014) take a multi-country approach on ageing and use information on Europeans aged 50 and older from the Survey of Health, Ageing and Retirement in Europe (SHARE). Achdut et al. (2014) use the same survey, but concentrate on respondents from a single country, Israel, to examine a number of specific features of the Israeli labour market and the effect of recent retirement reforms. The study by Tisch (2014), on the other hand, takes advantage of a rich administrative dataset from Germany with matched employer-employee information.

One of the main concerns with respect to prolonging employment is the argument that workers’ productivity declines over time. If this were the case, employing older workers would become unprofitable for employers if wages could not adjust downwards, and less attractive to the older employees if they could. While “age profiles” of earnings have been documented to have an inverse U-shape, it has been long recognised that there may be factors other than declining productivity, principally cohort effects and selection issues, that lie behind the seeming age-related decline (e.g. Myck 2010). There is, in fact, a growing body of evidence that age does not lead to lower productivity (Hellerstein et al. 1999; Mahlberg et al. 2013; Börsch-Supan and Weiss 2013). In fact, it has been demonstrated that productivity of teams including older workers may be greater compared to that of teams made up of only younger employees (Zwick and Göbel 2013). In a labour market which increasingly requires flexibility and adaptation of employees to changing requirements, productivity will undoubtedly depend on the ability to learn new skills and on the effectiveness of training. In this issue, Belloni and Villosio (2014) use information on past training activity and current wages in the SHARE survey for eleven European countries and find substantial cross-country differences in the level of “training premiums” for wages. Earlier training activity is associated with substantially higher wages after two years in Austria, Germany, Italy and Greece, while essentially no association is found in northern European countries and Switzerland. As the authors point out, the analysis may suffer from the usual challenge of pinning down the causal relationships (Goux and Maurin 2000) and the SHARE data cannot elicit the role of different types or intensities of training. However, the overall positive association between training and wages of over 6 percent combined with evidence from earlier studies on effectiveness of training (e.g. Pischke 2001; Dearden et al. 2006; Behaghel et al. 2014) is an argument for further, more detailed analysis of the causal nature of this relationship and of the identification of what types of training contribute best to productivity of older workers and why.

Apart from contributing to workers’ productivity and financial reward to work, training may also be an element of broader job quality, expressed through the potential for personal development and professional advancement. This, in turn, may be an important factor contributing to longer employment. As Dal Bianco et al. (2014) show in their article, low job quality significantly affects the desire to leave the labour market among older employees, and may also affect actual retirement decisions. Clearly, a complex combination of factors determine the decision to retire (Gustman and Steinmeier 1986; Blundell et al. 2002; Banks and Blundell 2005; Disney et al. 2006; Achdut et al. 2014), but it seems that the work environment, the “quality of a job”, is an arena with substantial potential for policy intervention. The important implications of Dal Bianco et al. (2014) are that improving work conditions, for example, through giving employees greater freedom and responsibilities at work or better stress management at work, could be a promising area for employers’ contribution to increases in older workers’ labour market activity and also a field for successful policy interventions.

Tisch (2014) uses the linked employer-employee dataset for Germany (LIAB) to look at job quality from a different perspective, namely to examine which of the employers’ characteristics correspond to a higher degree of employability of older workers. One of the key findings is that organisational characteristics of the employers are strongly related to internal employability, i.e. the ability to stay with a given employer. The employment sector and declared demand for skilled labour are the main drivers of this form of employability, with employers in services and those who look for skilled labour more likely to retain older workers. These organisational characteristics of the employer are less important for external employability, i.e. the individual probability of switching to another employer. As one would expect age is one of the main individual attributes which affects both internal and external employability. Education level is strongly correlated with the former, but not with the latter. Both aspects of employability are significant from the point of view of labour market policy. It seems, however, that identifying factors determining external employability and formulating policies to facilitate smooth transitions between jobs and re-employment of older workers will be a significant policy challenge. Job separations among older workers involve substantial losses of job-specific human capital and firm-specific “managerial culture”, and the hiring of older workers may be limited by deferred compensation schemes (Daniel and Heywood 2007; Heywood et al. 2010).

The rich set of information at the individual level in the SHARE dataset allows Achdut et al. (2014) to examine a similar set of transitions using the Israeli sample of the SHARE survey with a focus on detailed individual and household level characteristics. As has been demonstrated in a number of studies in the past (Disney et al. 2006; Haan and Myck 2009), health is confirmed to have a strong effect on the decision to leave employment, and higher wealth encourages individuals to retire earlier. As in Tisch (2014), higher age plays a key role in increasing the rate of employment exit and reduces the probability of returning to the labour force after exiting. The Israeli paper also shows interesting differences in labour market behaviour between different ethnic and religious groups. In addition, it provides descriptive evidence of effects of changes in the statutory retirement age, which were implemented in 2004 and were gradually introduced by 2010, i.e. mainly between the two analysed waves of the SHARE survey. This evidence adds to the body of literature on significant employment implications of the design of pensions systems and shows rapid labour market adjustments following their reforms (Börsch-Supan 2000).

Naturally, only a fraction of potential topics concerning determinants of labour market activity in older age are addressed in this special section, and a successful policy mix will have to take into account many other issues. Important policy challenges lie ahead with respect to unemployment benefits and welfare support, health policy and rehabilitation, disability benefit reforms, intergenerational knowledge transmission, and others. The four papers in this volume present, however, new evidence on some of the most important factors behind continued employment of older individuals and they stress the importance of some crucial aspects having policy relevance. Changes in the requirements of longer employment in the form of retirement system reforms (Achdut et al. 2014) will have to go hand in hand with policies supporting improvements in job quality (Dal Bianco et al. 2014) and appropriate training of older individuals (Belloni and Villosio 2014). The key challenge for policy, which will determine the success in these areas, will be how to successfully re-employ older workers after they leave or lose their jobs (Tisch 2014).
